# Functional *in silico* analysis of missense mutations in the MSH6 gene

**DOI:** 10.1186/1897-4287-13-S1-A10

**Published:** 2015-09-09

**Authors:** Katarzyna Gajdel, Grzegorz Kurzawski

**Affiliations:** 1 Pomeranian Medical University, Faculty of Medical Biotechnology, Szczecin, Poland; 2 Department of Genetics and Pathology, International Hereditary Cancer Center, Pomeranian Medical University, Szczecin, Poland

## 

Missense mutations of mismatch repair (MMR) genes – *MLH1, MSH2, MSH6* and *PMS2* – may (but do not necessarily) play a role in the etiology of the Lynch syndrome (LS) which is one of the hereditary cancer susceptibility syndromes. Pathogenic mutations in one of those genes result in a higher risk of developing cancer in colon, endometrium, small intestine, ovary, urinary tract, stomach or breast. One in ten patients with LS have a germline mutation in *MSH6*, and 40% of all mutations in that gene are single amino acid substitutions.

One of the elements taking part in the MMR process is the MSH6 protein which, along with MSH2, is a component of a protein dimer – MutSα. MMR function is critical in maintaining cellular DNA integrity. Loss of that function results in increased number of uncorrected mutations which may lead to malignant tissue transformation. This is why it is crucial to determine whether the detected change is located in a DNA sequence coding the MMR protein and if it impairs the repair function. Alas, the consequences of single amino acid substitution are often harder to evaluate than outcomes of deletions, insertions or nonsense mutations. Moreover, performing a functional and molecular analysis for each detected alteration would be too expensive and unrealistic. This is where the use of prediction software would be of assistance.

The intent of this thesis was to determine the pathogenic significance of a set of missense mutations using selected *in silico* analysis tools and to evaluate their utility in everyday use.

Twenty-one *MSH6* missense mutations were analyzed, three of them already classified as non-pathogenic polymorphisms, and two had confirmed pathogenic status. They were used accordingly as negative and positive controls.

Three online programs were used for the analysis of those mutations, which allowed to evaluate each substitution from a different angle. SIFT utilizes alignment of homologous sequences from variety of organisms to determine the conservation of the substitution site and thereby estimates the potential pathogenicity of a mutation. PolyPhen-2 prediction is based on the sequence, protein structure and position of each mutation. The last of the used programs was ESEfinder which tested the impact of a mutation on the presence of ESEs (Exonic Splicing Enhancers) in the examined sequence.

After performing the *in silico* analysis and confronting the results with data available in literature and the Internet databases, it was established that next to one of the changes confirmed as non-pathogenic polymorphisms, there are probably eight further non-pathogenic ones. Likewise, beside the two confirmed pathogenic alterations, in the analyzed set there were probably five additional pathogenic mutations. The last group is formed by substitutions that remain uncertain because of their ambiguous results.

Following the analysis of the results and available data, a conclusion was reached that the sole use of *in silico* prediction is not sufficient for a complete classification of the mutations, but they may be helpful for decision making in choosing or prioritizing the variants worth further analysis.

